# Harmonic-Reduced Bias Circuit for Ultrasound Transducers

**DOI:** 10.3390/s23094438

**Published:** 2023-04-30

**Authors:** Hojong Choi

**Affiliations:** Department of Electronic Engineering, Gachon University, 1342 Seongnam-daero, Sujeong-gu, Seongnam 13120, Republic of Korea; hojongch@gachon.ac.kr; Tel.: +82-31-750-5591

**Keywords:** harmonic-reduced bias circuit, ultrasound instrument, ultrasound transducer

## Abstract

The gain of class-C power amplifiers is generally lower than that of class-A power amplifiers. Thus, higher-amplitude input voltage signals for class-C power amplifiers are required. However, high-amplitude input signals generate unwanted harmonic signals. Therefore, a novel bias circuit was proposed to suppress the harmonic signals generated by class-C power amplifiers, which improves the output voltage amplitudes. To verify the proposed idea, the input harmonic signals when using a harmonic-reduced bias circuit (−61.31 dB, −89.092 dB, −90.53 dB, and −90.32 dB) were measured and were found to be much lower than those when using the voltage divider bias circuit (−57.19 dB, −73.49 dB, −70.97 dB, and −73.61 dB) at 25 MHz, 50 MHz, 75 MHz, and 100 MHz, respectively. To further validate the proposed idea, the pulse-echo measurements were compared using the bias circuits. The peak-to-peak echo amplitude and bandwidth of the piezoelectric transducer, measured when using a harmonic-reduced bias circuit (27.07 mV and 37.19%), were higher than those achieved with a voltage divider circuit (18.55 mV and 22.71%). Therefore, the proposed scheme may be useful for ultrasound instruments with low sensitivity.

## 1. Introduction

Among medical systems, ultrasound instruments are widely used in ambulances [1,2,3,4,5]. Compared to bench-top ultrasound instruments, which have a 100–240 V alternating current (AC) cord, other ultrasound instruments have a limited power supply [6,7]. Current ultrasound instruments have structures similar to those of laptop computers [8,9]. For example, the cooling fan of laptop computers must fit into a smaller structure than that of desktop computers, and laptop computers are restricted in their central processing unit (CPU) and graphic processing unit (GPU) capability owing to their limited power supply. The cooling fan must be controlled to minimize performance degradation due to the increased temperature caused by CPU and GPU operation [10,11,12,13]. Compared to laptop computers, ultrasound instruments have an even greater restriction in terms of high-power capability because power amplifiers must produce high-voltage signals to trigger piezoelectric transducers. In ultrasound instruments, power amplifiers are among the most critical sources of power consumption that need cooling fans [14,15,16]. Therefore, proper power amplifier design is crucial to achieve reasonable performance in ultrasound instruments.

Several nonlinear power amplifiers could be used to achieve efficient power amplifier design. In this paper, previous research is reviewed for different types of piezoelectric transducer applications. A class-D power amplifier was developed to generate 2 kW of output power, resulting in better signal quality with a power piezoelectric load [17,18,19]. A class-D amplifier was developed to generate a maximum output voltage of 125 V_rms_ for a dielectric elastomer transducer [20]. A 1010 kHz class-DE power amplifier with output power of 800 mW was designed to generate a piezoelectric ultrasound transducer that can be integrated with MRI machines [21,22]. The MRI coils could interact with external inductor components in the power amplifier such that the designed power amplifier is implemented without any inductor components [23,24]. A 41.27 kHz and 133.3 mW class-E inverter with an impedance converter was designed for a Langevin transducer [25]. A 28.11 kHz class-E resonant inverter with output voltage of 112 V_rms_ operating under the zero-voltage switching condition was designed [26]. 

One of the most important specifications of power amplifiers for ultrasound instruments is the output voltage, which is related to the sensitivity of the piezoelectric transducer; another is power consumption, which indicates the performance of the battery [27,28,29,30]. Nonlinear power amplifiers generate lower DC power consumption than linear power amplifiers [31,32,33]. Therefore, they might be more useful for wireless electronic systems with a limited power supply. However, they carry certain drawbacks such as a higher input voltage requirement [34,35,36]. To generate a high-amplitude input greater than 1 V_p-p_ for a nonlinear power amplifier, a digital-to-analog converter (DAC) must be used [37,38,39].

**Figure 1:**
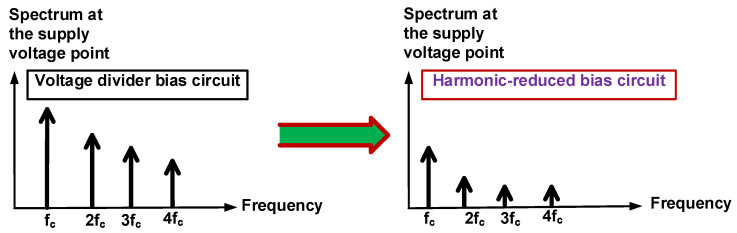
Proposed concept of a harmonic-reduced bias circuit for the class-C power amplifier.

Such high-amplitude input voltage can affect bias circuit stabilization and amplifier performance even when the power amplifier uses a shunt resistor or inductor to block unwanted input voltages, because the main transistors in the power amplifier must have a proper DC bias voltage and AC input signal simultaneously [40,41]. If an amplitude input voltage less than 1 V_p-p_ is the input of the power amplifier, more stage power amplifiers must be added because of the output of the amplifier [42,43]. Unfortunately, this could produce higher temperatures, thus possibly reducing the output performance during long-term operation [44]. Therefore, a new bias circuit to suppress unwanted input harmonic signals is proposed for use with high-amplitude input voltage. As depicted in Figure 1, a harmonic-reduced bias circuit could suppress several harmonic components of high-amplitude input signals (2f_c_, 3f_c_, and 4f_c_) simultaneously at the supply voltage point such that DC bias voltages could be more stabilized and more high-amplitude input signals go to the primary transistor for more effective amplification.

In most analog circuit design for power amplifiers, the analog passive filter in the output matching network is well known [45,46,47]. The passive filter in the output matching network affects some performance parameters of the power amplifier. However, the proposed approach could provide the harmonic-reduction capability in the bias circuit so that performance parameters such as bandwidth and linearity would be less affected [48]. With the proposed harmonic-reduced bias circuit, the voltage gain of the class-C power amplifier will be enhanced instead. 

If the passive filter approach in the output matching network is used, the specific harmonic distortion components could be reduced [49,50]. However, the voltage gain or bandwidth could be reduced accordingly. In particular, the high voltage generated by the power amplifier for ultrasound transducers could be more affected by the typical passive filters in the output matching network because the linear voltage amplification and wide bandwidth are essential due to the nonlinear ultrasound transducer load impedance [51,52,53]. 

Additionally, the variable resistors with low power levels in the passive filter could be hard to use because high-voltage output signals from power amplifiers for ultrasound applications are generated [54]. Compared to a typical passive filter in the output port of the power amplifier, the proposed harmonic-reduced bias circuit could be helpful to reduce the specific several harmonic distortion components in the echo signals generated by the ultrasound transducer. Especially in the ultrasound applications such as harmonic imaging, acoustic stimulation, and ultrasound therapy, harmonic signal reduction is more important than ultrasound imaging applications [55,56]. 

Unfortunately, simulation model libraries of high-voltage or high-power transistors used for power amplifier design have only sub-decibel-level distortion accuracy, so simulation data using high-voltage or high-power transistor models are inaccurate at predicting the voltage gain, power efficiency, and power consumption of power amplifiers [57,58,59]. A theoretical approach and experimental results are presented in this paper. In addition, the theoretical analysis of the harmonic-reduced bias circuit in the equivalent circuit model will be presented to predict how to design such a power amplifier. Section 2 presents detailed architecture, operating mechanisms, and equivalent circuit model analysis for a class-C power amplifier with a typical voltage divider and harmonic-reduced bias circuit. Section 3 shows the measurement results to characterize and evaluate the capability of harmonic signal compression with bias circuits. The voltage gain and power consumption performance of the class-C power amplifier with bias circuits including pulse-echo mode measurement were sufficient for ultrasound applications. Section 4 concludes the paper.

## 2. Materials and Methods

### 2.1. The Class-C Power Amplifier Fabrication

**Figure 2:**
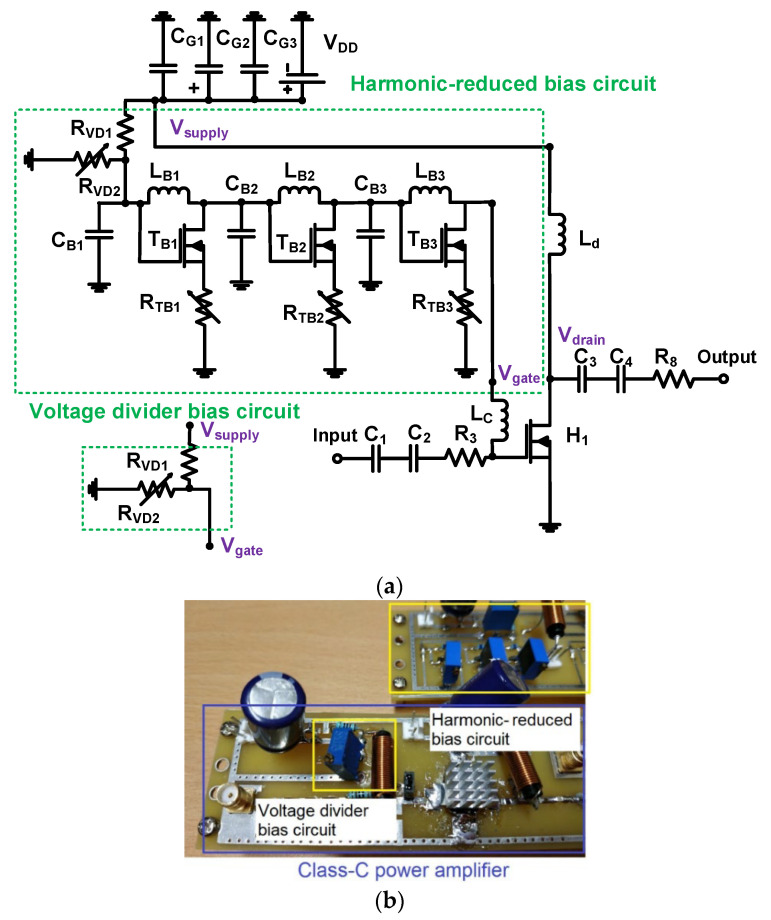
(**a**) Full schematic diagram and (**b**) fabricated class-C power amplifier with bias circuits on the printed circuit board.

Figure 2 shows a full schematic diagram and fabricated printed circuit board of the class-C power amplifier with harmonic-reduced and voltage divider bias circuits. The gate and drain sides in primary transistor (H_1_, ST Microelectronics, Inc., Geneva, Switzerland) were used by RF (radio frequency) choke inductors (L_c_ and L_d_, Bourns, Inc., Riverside, CA, USA) to reduce the DC voltage reduction of the class-C power amplifier [60]. The electrolytic capacitor (220 μF, Panasonic North America, Newark, NJ, USA) and additional ceramic capacitors (0.1 μF, 1 nF, and 0.1 nF, Vishay Siliconix, Santa Clara, CA, USA) were used to reduce possible high-frequency noises generated by the DC power supply [61,62]. The specification of the primary transistor (H_1_) is 1 GHz operating frequency, ±20 V gate-source voltage, and 65 V gate-drain voltage, which can be suitable for class-C power amplifier design. Two separate capacitors were used in the input port (C_1_ and C_2_, Bourns, Inc.) and output port (C_3_ and C_4_, Bourns, Inc.), respectively, because there is more freedom to select capacitance values when using two discrete capacitor components. Two resistors (R_3_ and R_8_) were used to smooth out the input and output waveforms.

The harmonic-reduced bias circuit is a kind of filter structure composed of inductors (L_B1_, L_B2_, and L_B3_, Coilcraft, Inc., Cary, IL, USA), capacitors (C_B1_, C_B2_, and C_B3_, Vishay Siliconix), transistors (T_B1_, T_B2_, and T_B3_, Diodes, Inc., Plano, TX, USA), and variable resistors (R_TB1_, R_TB2_, and R_TB3_, 10 kΩ, Bourns, Inc.) including one fixed resistor and one variable resistor (R_VD1_ and R_VD2_, 2 kΩ and 0.5 kΩ, Bourns, Inc.). The harmonic-reduced bias circuit is designed to block unwanted high-amplitude input signals with specific frequency components from the gate (V_gate_) to the supply voltage (V_supply_) and minimize the DC voltage reduction simultaneously because one RF choke inductor (L_c_) cannot completely block high-amplitude input signals higher than 1 V_p-p_. A voltage divider bias circuit that also has identical value resistors (R_VD1_ and R_VD2_, Bourns, Inc.) in a harmonic-reduced bias circuit was used to compare the bias circuit performances. Figure 2a,b give the schematic diagram and printed circuit board of the power amplifier integrated with harmonic-reduced and voltage divider bias circuit. 

The reason to use the voltage divider in the harmonic-reduced bias circuit is to compare the performance of the voltage divider bias circuit with that of the harmonic-reduced bias circuit. In the proposed harmonic-reduced bias circuit, it is possible to reduce certain harmonic distortion components such as second, third, and fourth harmonic distortion components simultaneously. The ultrasound transducer is a kind of capacitive load with resistance, capacitance, and inductance [63,64,65]. Therefore, the operating frequency in the second, third, and fourth harmonic distortion components of the power amplifier is not exactly two, three, and four times the operating frequency of the ultrasound transducer, respectively [66,67,68]. The specific harmonic distortion component technique can be controlled with the help of the proposed harmonic-reduced bias circuit. Therefore, the proposed approach could be helpful to improve the performance of certain ultrasound applications such as harmonic imaging, acoustic stimulation, and ultrasound therapy. 

### 2.2. Equivalent Circuit Analysis of Bias Circuits

**Figure 3:**
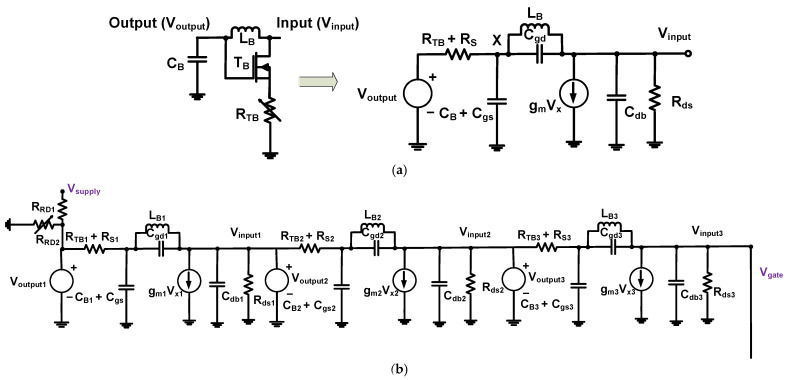
(**a**) One part of the harmonic-reduced bias circuit and (**b**) entire harmonic-reduced bias circuit with power supply resistor.

Figure 3 shows the equivalent circuit models of the bias circuits for AC analysis. The equivalent circuit models could describe the operating mechanisms of the harmonic-reduced and voltage divider bias circuits. In the equivalent circuit model of the transistor (Figure 3a), the drain–source resistance was removed because its value in the transistor was very small. Therefore, the equivalent circuit model of the transistor had parasitic gate–source, gate–drain, and drain–source capacitances (C_gs_, C_gd_, and C_ds_) [69,70,71,72]. 

Figure 3a shows the equivalent circuit model of only one part of the harmonic-reduced bias circuit except for the resistors (R_RD1_ and R_RD2_). We expected that unwanted high-amplitude input signals would come from the gate of the transistor, so the input and output voltages (V_input_ and V_output_) were labeled in opposite directions. 

As shown in Figure 3a, one part of the equivalent circuit in the harmonic-reduced bias circuit can be constructed. Using the equivalent circuit model as shown in Figure 3a, the voltage (V_x_) at each node could be calculated via Equations (1) and (2) [73,74,75]:

(1) $$\frac{V_{X}-V_{\mathrm{output}}}{R_{\mathrm{TB}}+R_{S}}+V_{X}\left(C_{B}+C_{\mathrm{gs}}\right)j2{\pi f}_{c}+\frac{\left(V_{X}-V_{\mathrm{input}}\right)}{\left(\frac{1}{j2{\pi f}_{c}C_{\mathrm{gd}}}∥j2{\pi f}_{c}L_{B}\right)}=0$$

(2) $$\frac{\left(V_{\mathrm{input}}-V_{X}\right)}{\left(\frac{1}{j2{\pi f}_{c}C_{\mathrm{gd}}}∥j2{\pi f}_{c}L_{B}\right)}+g_{m}V_{X}+V_{\mathrm{input}}(\frac{1}{R_{\mathrm{ds}}}+j2{\pi f}_{c}C_{\mathrm{db}})=0\;,\;$$

where R_s_ is the source resistance, C_gs_ is the gate-source capacitance, C_gd_ is the gate-drain capacitance, f_c_ is the center frequency, C_db_ is the drain capacitance, g_m_ is the transconductance, R_ds_ is the drain-source resistance, C_B_ is the shunt capacitance, and L_B_ is the series inductance.

Combining Equations (1) and (2), the voltage (V_x_) could be obtained as per Equation (3):

(3) $$V_{X}=-V_{\mathrm{input}}\frac{\left(\left(\frac{1}{j2{\pi f}_{c}C_{\mathrm{gd}}}∥j2{\pi f}_{c}L_{B}\right)+\frac{1}{R_{\mathrm{ds}}}+j2{\pi f}_{c}C_{\mathrm{db}}\right)}{g_{m}-\left(\frac{1}{j2{\pi f}_{c}C_{\mathrm{gd}}}∥j2{\pi f}_{c}L_{B}\right)}\;.$$

After the voltage (V_x_) in Equation (3) is applied to Equation (1), Equation (4) could be obtained:

(4) $$-V_{\mathrm{input}}\frac{\left[\frac{1}{R_{\mathrm{TB}}+R_{S}}+\left(\frac{1}{j2{\pi f}_{c}\left(C_{B}+C_{\mathrm{gs}}\right)}+\left(\frac{1}{j2{\pi f}_{c}C_{\mathrm{gd}}}∥j2{\pi f}_{c}L_{B}\right)\right)\right]\left[\frac{1}{R_{\mathrm{ds}}}+\left(\frac{1}{j2{\pi f}_{c}C_{\mathrm{gd}}}∥j2{\pi f}_{c}L_{B}\right)+\frac{1}{j2{\pi f}_{c}C_{\mathrm{db}}}\right]}{g_{m}-\left(\frac{1}{j2{\pi f}_{c}C_{\mathrm{gd}}}∥j2{\pi f}_{c}L_{B}\right)}\;-V_{\mathrm{input}}\left(\frac{1}{j2{\pi f}_{c}C_{\mathrm{gd}}}∥j2{\pi f}_{c}L_{B}\right)=\frac{V_{\mathrm{output}}}{R_{\mathrm{TB}}+R_{S}}.$$

The transfer function of one equivalent circuit of the harmonic-reduced bias circuit is represented in Equation (5):

(5) $$\frac{V_{\mathrm{input}}}{V_{\mathrm{output}}}=\left[(R_{\mathrm{TB}}+R_{S}\right)(\frac{\left[\frac{1}{R_{\mathrm{TB}}+R_{S}}+\left(\frac{1}{j2{\pi f}_{c}\left(C_{B}+C_{\mathrm{gs}}\right)}+\left(\frac{1}{j2{\pi f}_{c}C_{\mathrm{gd}}}∥j2{\pi f}_{c}L_{B}\right)\right)\right]\left[\frac{1}{R_{\mathrm{ds}}}+\left(\frac{1}{j2{\pi f}_{c}C_{\mathrm{gd}}}∥j2{\pi f}_{c}L_{B}\right)+\frac{1}{j2{\pi f}_{c}C_{\mathrm{db}}}\right]+\left(\frac{1}{j2{\pi f}_{c}C_{\mathrm{gd}}}∥j2{\pi f}_{c}L_{B}\right)}{g_{m}-\left(\frac{1}{j2{\pi f}_{c}C_{\mathrm{gd}}}∥j2{\pi f}_{c}L_{B}\right)}){]}^{-1}\;.$$

An unwanted high-amplitude input signal was passed through one part of the harmonic-reduced bias circuit from the “input” (V_input_) to “output” (V_output_) ports. This worked as a kind of filter [76,77,78]. Figure 3b shows the equivalent circuit model of the harmonic-reduced bias circuit with the resistance of the power supply (R_supply_). To minimize several higher-harmonic components of the input signal, three succeeding circuits were adopted in the harmonic-reduced bias circuit as shown in Figure 3b. The transfer function of three filter circuits of the harmonic-reduced bias circuit with the power supply resistance can be obtained via Equation (6):

(6) $$\frac{V_{\mathrm{supply}}}{V_{\mathrm{gate}}}=\frac{V_{\mathrm{input}1}}{V_{\mathrm{output}1}}\cdot \frac{V_{\mathrm{input}2}}{V_{\mathrm{output}2}}\cdot \frac{V_{\mathrm{input}3}}{V_{\mathrm{output}3}}\left[1+\frac{R_{\mathrm{supply}}}{R_{\mathrm{VD}1}}\right],\;$$

where V_input1_, V_input2_, and V_input3_ are the first, second, and third inputs of the harmonic-reduced bias circuit, respectively. V_output1_, V_output2_, and V_output3_ are the first, second, and third outputs of the harmonic-reduced bias circuit, respectively.

**Figure 4:**
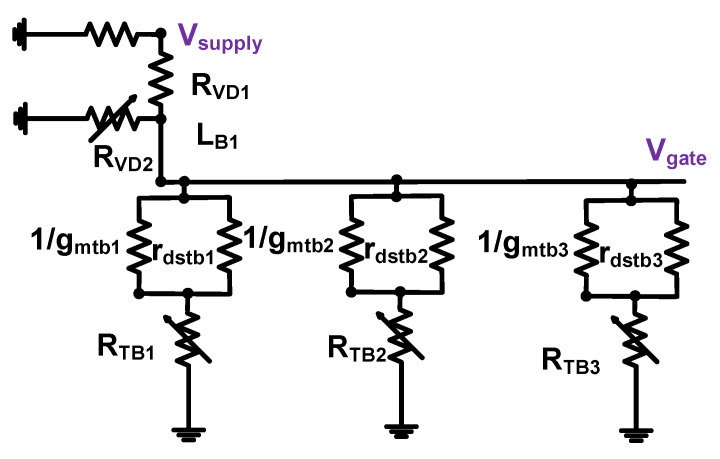
Equivalent circuit model of the harmonic-reduced bias circuit for DC analysis.

Figure 4 shows the equivalent circuit model of the harmonic-reduced bias circuit for DC analysis.

According to the voltage divider theory, the DC voltage of the gate (V_gate_) can be simply represented by Equation (7) [79]. The values of three shunt resistances (R_TB1_, R_TB2,_ and R_TB3_) are much larger than the values of the parallel impedance of the transconductances (g_mtb1_, g_mtb2_, and g_mtb3_) and drain-source resistances (r_dstb1_, r_dsb2_, and r_dsb3_) of the transistors, so we can ignore those values. If three shunt resistances (R_TB1_, R_TB2_, and R_TB3_) are much larger than one shunt resistance (R_VD2_), the combinational resistances (R_VD2_, R_TB1_, R_TB2_, and R_TB3_) could be simplified into one shunt resistance (R_VD2_), as shown in Equation (8). Thus, the DC voltage of the gate in the harmonic-reduced and voltage divider bias circuits (V_gate_) could be similar. As shown in Equation (8), three shunt resistances were not affected accordingly. Consequently, the harmonic-reduced bias circuit could suppress the unwanted high-amplitude input signal while sustaining the same DC bias voltages between the voltage divider and harmonic-reduced bias circuits if the shunt resistors (R_TB1_, R_TB2_, and R_TB3_) are much larger than one shunt resistor (R_VD2_).

(7) $$V_{\mathrm{gate}}=(\frac{R_{\mathrm{VD}2}∥(R_{\mathrm{TB}1}+\frac{1}{g_{\mathrm{mtb}1}}∥r_{\mathrm{dstb}1})∥(R_{\mathrm{TB}2}+\frac{1}{g_{\mathrm{mtb}2}}∥r_{\mathrm{dstb}2})∥(R_{\mathrm{TB}3}+\frac{1}{g_{\mathrm{mtb}3}}∥r_{\mathrm{dstb}3})}{R_{\mathrm{VD}1}+R_{\mathrm{VD}2}∥(R_{\mathrm{TB}1}+\frac{1}{g_{\mathrm{mtb}1}}∥r_{\mathrm{dstb}1})∥(R_{\mathrm{TB}2}+\frac{1}{g_{\mathrm{mtb}2}}∥r_{\mathrm{dstb}2})∥(R_{\mathrm{TB}3}+\frac{1}{g_{\mathrm{mtb}3}}∥r_{\mathrm{dstb}3})})V_{\mathrm{supply}}\;=(\frac{1}{1+\frac{R_{\mathrm{VD}1}}{R_{\mathrm{VD}2}∥R_{\mathrm{TB}1}∥R_{\mathrm{TB}2}∥R_{\mathrm{TB}3}}})V_{\mathrm{supply}}$$

(8) $$V_{\mathrm{gate}}\approx (\frac{1}{1+\frac{R_{\mathrm{VD}1}}{R_{\mathrm{VD}2}}}){\;V}_{\mathrm{supply}}=(\frac{R_{\mathrm{VD}2}}{R_{\mathrm{VD}1}+R_{\mathrm{VD}2}}){\;V}_{\mathrm{supply}}$$

The relationship between the i_output_ and V_gate_ of the class-C power amplifier with temperature effect can be represented by Equation (9) [80,81]:

(9) $$i_{\mathrm{output}}=\frac{I_{\mathrm{drain}}V_{\mathrm{drain}}}{\pi \cdot (V_{\mathrm{gate}}-\frac{\sqrt{2{\mathrm{qN}}_{A}\varepsilon \left(2\Phi_{f}\right)}}{C_{\mathrm{OX}}}+2\Phi_{f}+\Phi_{\mathrm{ms}}-\frac{Q_{\mathrm{ss}}}{C_{\mathrm{OX}}})}\left[\theta_{\mathrm{conduction}}-\sin\left(\theta_{\mathrm{conduction}}\right)\right],\;$$

where q is the charge, N_A_ is the constant doping density of the p-type substrate, ε is the permittivity of the oxide, Φ_f_ is the Femi level, C_ox_ is the oxide capacitance per unit, Φ_f_ is the work function difference between the oxide and silicon interface, Q_ss_ is the positive charge density in the oxide at the silicon interface, and θ_conduction_ is the conduction angle.

The gain and power consumption of the class-C power amplifier with harmonic-reduced and voltage divider bias circuits are apparently the same, except for the conduction angle (θ_conduction_) and output current (i_output_) values, owing to different DC bias voltages. The voltage gain (G_C_) of the class-C power amplifier with bias circuits can be expressed by Equation (10) [82,83]:

(10) $$G_{C}=i_{\mathrm{output}}\cdot \frac{R_{\mathrm{Load}}}{2\pi \cdot V_{\mathrm{input}}}\left(2\theta_{\mathrm{conduction}}-\sin2\theta_{\mathrm{conduction}}\right),\;$$

where I_drain_, θ_conduction_, and V_drian_ are the drain current, conductance angle, and drain voltage of the transistor. R_Load_ is the load impedance of the oscilloscope, and i_output_ and V_input_ are the output current and input voltage of the class-C power amplifier with bias circuits, respectively

The static and dynamic power consumption (P_C_) of the class-C power amplifier with bias circuits is represented in Equation (11) [84,85,86]. In a class-A power amplifier, power consumption can be obtained by multiplying the supply voltage by the output current. However, the class-C power amplifier depends on the conduction angle (θ_conduction_) and dynamic power consumption [87].

(11) $$P_{C}=V_{\mathrm{supply}}\left[\frac{i_{\mathrm{output}}}{\pi }\left({\sin\theta }_{\mathrm{conduction}}-\theta_{\mathrm{conduction}}{\cos\theta }_{\mathrm{conduction}}\right)\right]+C_{p}V_{\mathrm{DD}}{}^{2}f_{c},\;$$

where C_p_ is the dynamic power-dissipation capacitance. 

The next section shows the measurement results of the designed circuits such as spectrum data in the input port to show the effects of the input signal waveform. The gain and power consumption were measured to estimate the performance of the power amplifier. In addition, the pulse-echo responses using the ultrasound transducer and designed circuits were shown.

The transistor model used for the power amplifier has only sub-decibel level distortion accuracy, so that the simulation data are inaccurate at predicting the voltage gain and power consumption [80]. In addition, there are no simulation libraries for variable resistors, transistors, electrostatic capacitors, RF inductors, and choke inductors. 

## 3. Results and Discussion

### 3.1. Performance Evaluation of the Bias Circuits

**Figure 5:**
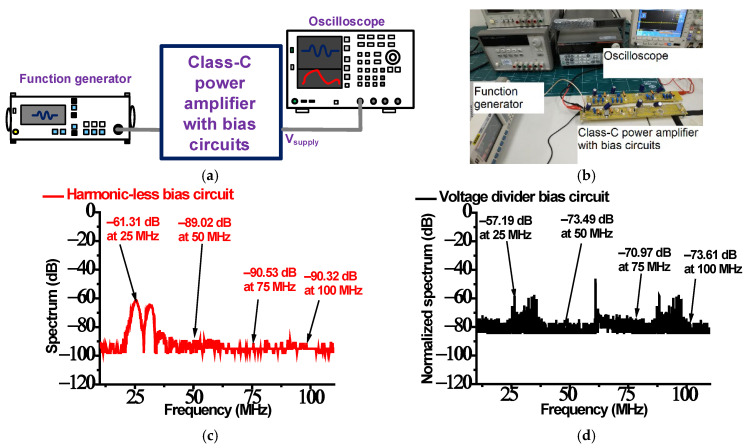
(**a**) Schematic diagram and (**b**) photo of measurement setup; measured output spectrum for (**c**) harmonic-reduced and (**d**) voltage divider bias circuits.

The measured performance of the harmonic-reduced bias circuit is provided in Figure 5. Figure 5a,b show the measurement setup to validate the capability of the harmonic-reduced bias circuit. The unwanted high-amplitude input voltage blocking performance was compared between the implemented bias circuits. A 25 MHz, 5-cycle, and 5 V_p-p_ sinusoidal waveform from a function generator (DG5071, Rigol Technologies, Inc., Beijing, China) was the input, and the output voltage (V_supply_) was recorded as spectrum data with an oscilloscope (MSO2024B, Tecktronics, Inc., Beaverton, OR, USA). 

Figure 5c,d show the spectrum data of the output ports measured at the supply point (V_supply_). In Figure 5c, the spectrum data measured when using the harmonic-reduced bias circuit at the center frequency (25 MHz), second harmonic (50 MHz), third harmonic (75 MHz), and fourth harmonic (100 MHz) components were −61.31 dB, −89.02 dB, −90.53 dB, and −90.32 dB, respectively. In Figure 5d, the spectrum data measured when using the voltage divider bias circuit at the center frequency (25 MHz), second harmonic (50 MHz), third harmonic (75 MHz), and fourth harmonic (100 MHz) components were −57.19 dB, −73.49 dB, −70.97 dB, and −73.61 dB, respectively. The unwanted amplitudes at the center frequency, second, third, and fourth harmonic components could be resonated out to be filtered. This measured performance would prove this theory.

The harmonic-reduced bias circuit showed better harmonic signal suppression than the voltage divider bias circuit at all harmonic signal components. Therefore, this capability can help improve class-C power amplifier performance because more input signal amplitudes may go to the primary transistor. In the next section, the gain and power consumption performance were measured to further validate our proposed idea. Table 1 summarizes the measured results of the harmonic-reduced and voltage divider bias circuits.

### 3.2. Power Amplifier Performance Evaluation

**Figure 6:**
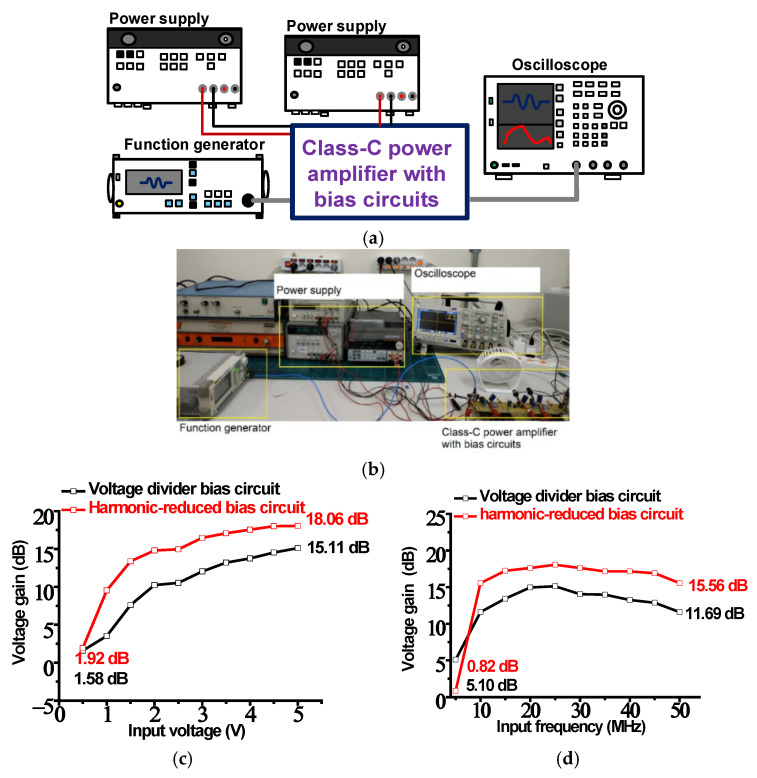

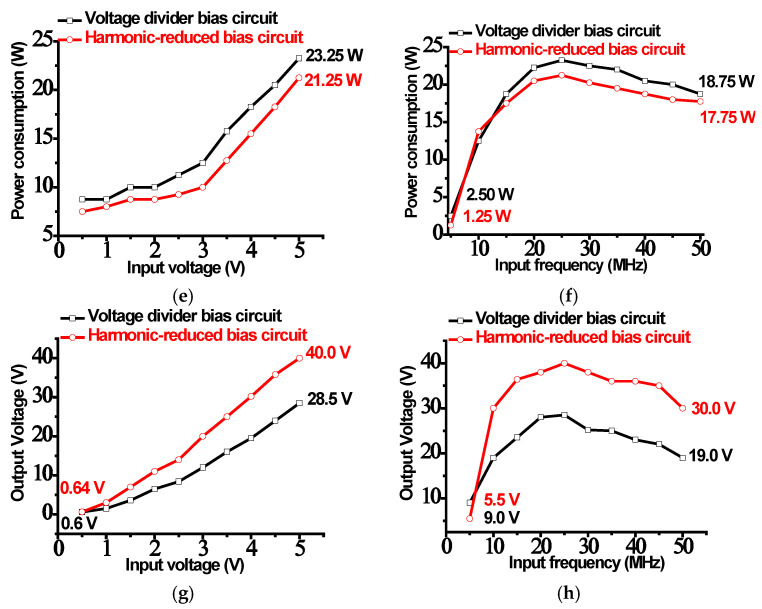
(**a**) Schematic diagram and (**b**) photo of measurement setup; the gain versus (**c**) input voltage and (**d**) input frequency of the class-C power amplifier with bias circuits; the power consumption versus (**e**) input voltage and (**f**) input frequency of the class-C power amplifier with bias circuits; The output voltage versus (**g**) input voltage and (**h**) input frequency of the class-C power amplifier with bias circuits.

The gain of the power amplifier is related to the echo signal sensitivity, and the power consumption is related to the instrument battery [88,89]. Therefore, the parameters of the power amplifiers could be the output voltage and power consumption. The gain and power consumption of the class-C power amplifier with bias circuits are presented accordingly. Figure 6a,b show the schematic diagram and photo of the measurement setup for the gain and power consumption of the class-C power amplifiers with bias circuits. A 25 MHz and 5-cycle input waveform generated from a function generator (DG5071) was used as the input of the class-C power amplifier with bias circuits. The output voltage was obtained in the oscilloscope (MSO2024B), and the gain was calculated by dividing the output voltage by the input voltage. 

Figure 6c,d show the measured gain versus input voltage and input frequency of the class-C power amplifier with bias circuits. In Figure 6c, the maximum gain when using the class-C power amplifier with the harmonic-reduced bias circuit (18.06 dB) was higher than that when using the class-C power amplifier with the voltage divider bias circuit (15.11 dB) at a 5 V_p-p_ input voltage. In Figure 6d, the minimum gain when using the class-C power amplifier with the harmonic-reduced bias circuit (0.82 dB) was lower than that when using the class-C power amplifier with the voltage divider bias circuit (5.10 dB) at a 5 MHz input frequency. However, the maximum gain when using the class-C power amplifier with the harmonic-reduced bias circuit (15.56 dB) was higher than that when using the class-C power amplifier with the voltage divider bias circuit (11.69 dB) at a 50 MHz input frequency. 

Figure 6e,f show the measured power consumption of the class-C power amplifier with the bias circuits versus the input voltage and input frequency. The power consumption performance is an important parameter for ultrasound instruments because of the battery issue. In Figure 6e, the measured power consumption of the class-C power amplifier with the harmonic-reduced bias circuit (21.25 W) was less than that of the class-C power amplifier with the voltage divider circuit (23.25 W) versus the input voltage at 5 V. In Figure 6f, the measured power consumption of the class-C power amplifier with the harmonic-reduced bias circuit (17.75 W) was less than that of the class-C power amplifier with the voltage divider circuit (18.75 W) versus the input frequency at 50 MHz. Figure 6g shows the measured output voltage versus the input voltage of the class-C power amplifier with the bias circuits. In Figure 6g, the output voltage of the class-C power amplifier with the harmonic-reduced bias circuit (40.0 V) was higher than that of the class-C power amplifier with the voltage divider circuit (28.5 V) versus the input voltage at a 5 V input voltage. Figure 6h shows the measured output voltage versus the input frequency of the class-C power amplifier with the bias circuits. In Figure 6h, the output voltage of the class-C power amplifier with the harmonic-reduced bias circuit (30.0 V) was higher than that of the class-C power amplifier with the voltage divider circuit (19.0 V) versus the input voltage at a 50 MHz input frequency.

Table 2 shows the measured gain and power consumption versus input voltage and input frequency of the class-C power amplifiers with harmonic-reduced and voltage divider bias circuits, respectively.

Table 3 shows comparison data of the maximum gain and power consumption versus input voltage and input frequency of the class-C power amplifiers with harmonic-reduced and voltage divider bias circuits, respectively. The maximum gain of the harmonic-reduced bias circuit was higher than that of the voltage divider circuit. In addition, the maximum power consumption of the harmonic-reduced bias circuit was lower than that of the voltage divider circuit. These results confirm that the proposed harmonic-reduced bias circuits could reduce the effects of the high-voltage input signals on the bias points of the main transistor in the class-C power amplifiers.

### 3.3. Pulse-Echo Mode Measurement

**Figure 7:**
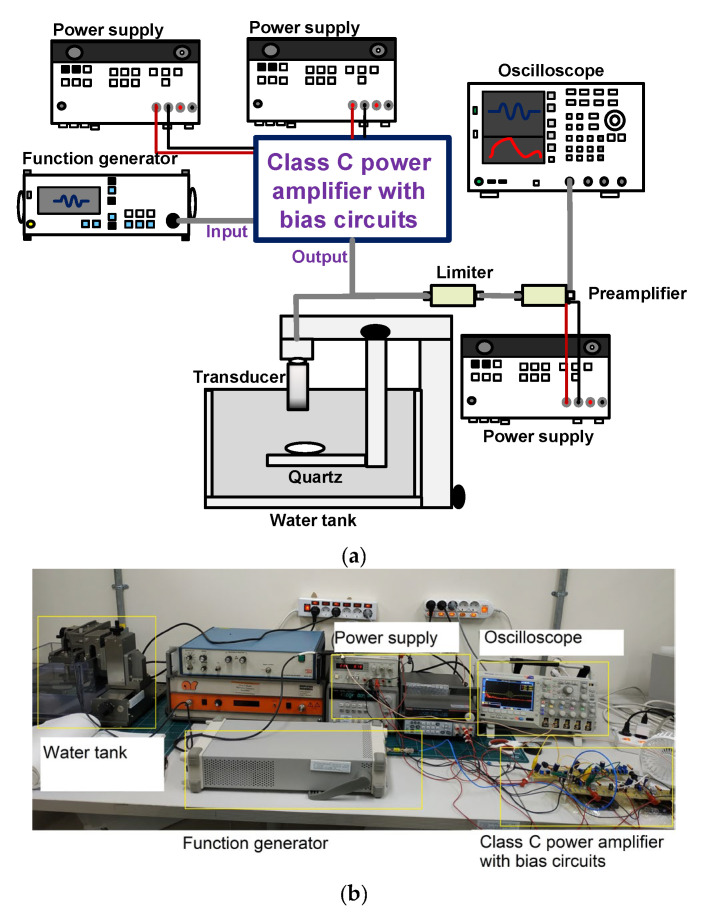
(**a**) Schematic diagram and (**b**) photo of the pulse-echo mode measurement.

Pulse-echo measurement is a basic operating method for evaluating piezoelectric transducers and electronics [90,91,92]. Figure 7a,b show the schematic diagram and photo of the pulse-echo measurement setup to evaluate the performance of the class-C power amplifier with bias circuits. A 25 MHz, 5-cycle, and 5 V_p-p_ sinusoidal waveform from a function generator was used as the input. The input was fed into the developed class-C power amplifier with bias circuits. The generated pulses were transmitted into a piezoelectric transducer to produce the acoustic pulses to be delivered to the target, and the discharged pulses were blocked by a limiter comprising a resistor shunt with a single cross-coupled diode [93]. The echoes were detected by a piezoelectric transducer with the element size of 0.25 inch and operating frequency of 25 MHz converted into the echo waveform [9]. The weak echo waveforms were amplified by a preamplifier (AU-1114, MITEQ, Inc., Hauppauge, NJ, USA), and the waveform and its spectrum were displayed on an oscilloscope (MSO2024B). The peak-to-peak voltage (V_p-p_), center frequency (f_c_), −6 dB BW (bandwidth), and total harmonic distortion (THD) of the measured echo signal were calculated using Equations (12)–(15) [94,95,96,97]:

(12) $$V_{p-p}=V_{+}-V_{-}$$

(13) $$f_{c}=\frac{f_{c1}+f_{c2}}{2}$$

(14) $$-6\;\mathrm{dB}\;\mathrm{BW}=\left(\frac{f_{c2}-f_{c1}}{f_{c}}\right)\cdot 100$$

(15) $$\mathrm{THD}=20\mathrm{Log}\frac{\left[{\left(V_{2}\right)}^{2}+{\left(V_{3}\right)}^{2}+{\left(V_{4}\right)}^{2}\right]}{V_{1}{}^{2}},$$

where V_+_ and V_−_ were the measured positive maximum and negative minimum voltages, respectively; f_c1_ and f_c2_ were measured frequency range located at the left and right points −6 dB below the frequency at maximum spectrum data; V_1_ is the amplitude of the fundamental signal; and V_2_, V_3_, and V_4_ are the amplitudes of the second, third, and fourth harmonic signals, respectively.

**Figure 8:**
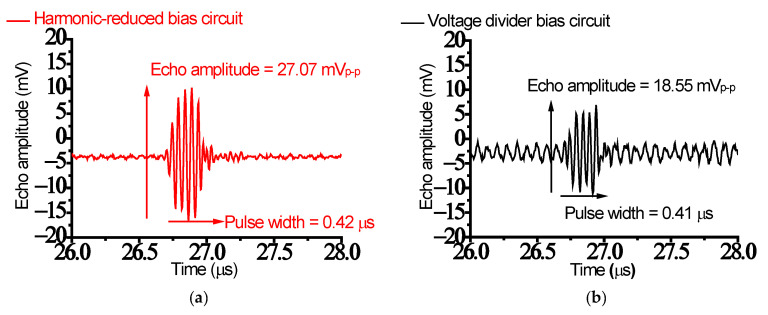

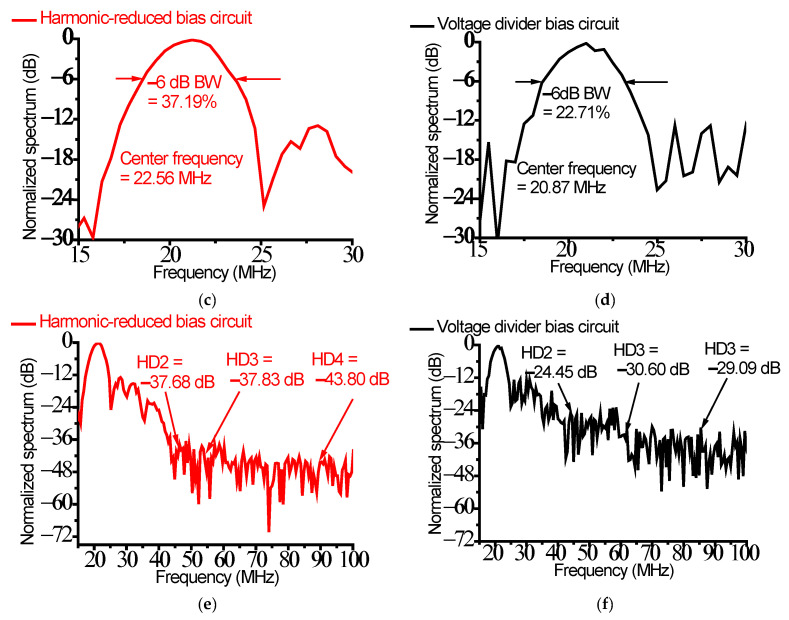
Echo signal amplitude when using (**a**) harmonic-reduced and (**b**) voltage divider bias circuits; echo signal spectrum when using (**c**) harmonic-reduced and (**d**) voltage divider bias circuits; enlarged echo signal spectrum to show the harmonic distortions (HD2, HD3, and HD4) when using (**e**) harmonic-reduced and (**f**) voltage divider bias circuits.

Figure 8a,b show the echo signal amplitude comparison measured when using different bias circuits. The measured echo signal amplitude when using the harmonic-reduced bias circuit (27.07 mV_p-p_) was higher than that when using the voltage divider bias circuit (18.55 mV_p-p_) because of the higher gain measured when using the harmonic-reduced bias circuit. Figure 8c,d show the comparison of the echo signal spectrum measured when using different bias circuits. The center frequency measured when using the harmonic-reduced bias circuit (22.56 MHz) was higher than that when using the voltage divider bias circuit (20.87 MHz). The −6 dB BW measured when using the harmonic-reduced bias circuit (37.19%) was also larger than that when using the voltage divider bias circuit (22.71%). 

Figure 8e,f show enlarged normalized spectrum data of Figure 8c,d to compare the harmonic distortion signals (HD2, HD3, and HD4). The harmonic distortion signals (HD2 = −37.68 dB, HD3 = −37.83 dB, and HD4 = −43.80 dB) when using the harmonic-reduced bias circuit were lower than those (HD2 = −24.45 dB, HD3 = −30.60 dB, and HD4 = −29.09 dB) when using the voltage divider circuit. The calculated THD value (−34.82 dB) when using the harmonic-reduced bias circuit was also lower than that when using the voltage divider circuit. These harmonic distortion data confirm that the proposed harmonic-reduced bias circuit could affect the signal distortions of the echo signals generated by the ultrasound transducer.

Table 4 summarizes the measured comparison data of the echo signal amplitude and spectrum data when using the harmonic-reduced and voltage divider bias circuits. The class-C power amplifier with the harmonic-reduced bias circuit may be a better candidate for reducing signal distortions in electronic devices.

Table 5 shows the comparison data of the proposed work with previous publications related to the nonlinear power amplifiers only for ultrasound applications because the designed scheme is useful for nonlinear power amplifiers that have large harmonic distortions. Therefore, the linear power amplifier schemes were excluded in Table 5 as below. Their target applications are piezoelectric transducer applications, even though their design circuit topology is different. Therefore, comparison data are provided here. 

The target applications of the other power amplifier design are low-frequency (≤15 MHz) ultrasound transducers. Compared to other previous publications, the target application of the designed class-C power amplifier with bias circuits is a high-frequency (≥15 MHz) piezoelectric transducer. Because the designed bias circuit to suppress harmonic signals from high-amplitude input signals was proposed, the harmonic distortion performance needs to be properly produced with the help of the designed harmonic-reduced bias circuit. The HD3 (−37.83 dB) when using the proposed bias circuit of the class-C power amplifier was measured.

## 4. Conclusions

Ultrasound instruments are used in hospitals. Compared to bench-top ultrasound instruments, other ultrasound instruments have the advantage of being cordless for portability. However, their performance is severely restricted by power consumption owing to battery limitations. Because thermal problems could affect the performance of ultrasound instruments, the cooling fan must be utilized, resulting in unavoidable noise, which affects the image resolution of the ultrasound instruments. To solve this problem, nonlinear power amplifiers would be more desirable than linear power amplifiers owing to their low power consumption. However, nonlinear power amplifiers may produce lower output power owing to the low conduction angle of the main transistors. Consequently, a higher input amplitude is required to generate adequate echo signal outputs from the ultrasound transducers. 

To utilize the class-C power amplifiers in the ultrasound instruments, high-amplitude (>1 V_p-p_) input voltage signals must be applied to obtain proper output voltages, because the gain of the class-C power amplifiers is lower than that of the class-A power amplifier. Therefore, a new type of harmonic-reduced bias circuit was proposed to block unwanted high-amplitude input signals. To verify the capability of the bias circuit, the measured spectrum data at a DC supply voltage point were measured. The measured spectrum of the harmonic-reduced bias circuit was lower (−61.31 dB, −89.02 dB, −90.53 dB, and −90.32 dB) than that of the voltage divider bias circuit (−57.19 dB, −73.49 dB, −70.97 dB, and −73.61 dB) at 25 MHz, 50 MHz, 75 MHz, and 100 MHz, respectively. The measured gain of the class-C power amplifier with the harmonic-reduced bias circuit was higher (18.06 dB) than that of the class-C power amplifier with the voltage divider circuit (15.11 dB). The measured power consumption when using the class-C power amplifier with the harmonic-reduced bias circuit (21.25 W) was lower than that when using the class-C power amplifier with the voltage divider circuit (23.25 W). 

To further verify the capabilities of the harmonic-reduced bias circuit, a pulse-echo measurement was also performed. The echo signal amplitude and bandwidth measured when using the class-C power amplifier with the harmonic-reduced bias circuit (27.07 mV and 37.19%) were higher than those of the class-C power amplifier with the voltage divider circuit (18.55 mV and 22.71%). The THD value (−34.82 dB) calculated when using the class-C power amplifier with the harmonic-reduced bias circuit was lower than that when using the class-C power amplifier with the voltage divider circuit. These data confirm that the proposed harmonic-reduced bias circuit could help reduce the signal distortions of the echo signals. Therefore, the designed class-C power amplifier with a harmonic-reduced bias circuit could be a candidate to reduce harmonic distortion in transducer applications.

## Figures and Tables

**Table 1:** Comparison data of the measured output spectrum of the designed harmonic-reduced and voltage divider bias circuits.

	25 MHz	50 MHz	75 MHz	100 MHz
Harmonic-reduced Bias Circuit	−61.31 dB	−89.02 dB	−90.53 dB	−90.32 dB
Voltage Divider Bias Circuit	−57.19 dB	−73.49 dB	−70.97 dB	−73.61 dB

**Table 2:** Measured results of the gain and power consumption versus input voltage or input frequency of the designed power amplifiers with harmonic-reduced and voltage divider bias circuits, respectively.

	**Voltage Divider Bias Circuit**	**Harmonic-Reduced Bias Circuit**		**Voltage Divider Bias Circuit**	**Harmonic-Reduced Bias Circuit**
Input Voltage	Gain		Input Frequency	Gain	
0.5 V	1.58 dB	1.92 dB	5 MHz	5.10 dB	0.82 dB
1.0 V	3.52 dB	9.54 dB	10 MHz	11.59 dB	15.56 dB
1.5 V	7.60 dB	13.38 dB	15 MHz	13.44 dB	17.24 dB
2.0 V	10.23 dB	14.80 dB	20 MHz	14.96 dB	17.61 dB
2.5 V	10.52 dB	14.96 dB	25 MHz	15.11 dB	18.06 dB
3.0 V	12.04 dB	16.47 dB	30 MHz	14.04 dB	17.61 dB
3.5 V	13.20 dB	17.07 dB	35 MHz	13.97 dB	17.14 dB
4.0 V	13.76 dB	17.55 dB	40 MHz	13.25 dB	17.14 dB
4.5 V	14.53 dB	18.01 dB	45 MHz	12.86 dB	16.90 dB
5.0 V	15.11 dB	18.06 dB	50 MHz	11.59 dB	15.56 dB
	**Voltage Divider Bias Circuit**	**Harmonic-reduced Bias Circuit**		**Voltage Divider Bias Circuit**	**Harmonic-reduced Bias Circuit**
Input Voltage	Power Consumption		Input Voltage	Power Consumption	
0.5 V	8.75 W	7.50 W	5 MHz	2.50 W	1.250 W
1.0 V	8.75 W	8.00 W	10 MHz	12.50 W	13.75 W
1.5 V	10.00 W	8.75 W	15 MHz	18.75 W	17.50 W
2.0 V	10.00 W	8.75 W	20 MHz	22.25 W	20.50 W
2.5 V	11.25 W	9.25 W	25 MHz	23.25 W	21.25 W
3.0 V	12.50 W	10.00 W	30 MHz	22.50 W	20.25 W
3.5 V	15.75 W	12.75 W	35 MHz	22.00 W	19.50 W
4.0 V	18.25 W	15.50 W	40 MHz	20.50 W	18.75 W
4.5 V	20.50 W	18.25 W	45 MHz	20.00 W	18.00 W
5.0 V	23.25 W	21.25 W	50 MHz	18.75 W	17.75 W

**Table 3:** Comparison data of the measured results of the gain and power consumption of the designed power amplifiers with harmonic-reduced and voltage divider bias circuits.

	Gain vs. Input Voltage	Gain vs. Input Frequency	Power Consumption vs. Input Voltage	Power Consumption vs. Input Voltage
Harmonic-reduced Bias Circuit	18.06 dB at 5 V	15.56 dB at 50 MHz	21.25 W at 5 V	17.75 W at 50 MHz
Voltage Divider Bias Circuit	15.11 dB at 5 V	11.59 dB at 50 MHz	23.25 W at 5 V	18.75 W at 50 MHz

**Table 4:** Summary of the echo signal amplitude, −6 dB BW, HD2, HD3, HD4, and THD data.

	Amplitude	Pulse Width	−6 dB BW	HD2	HD3	HD4	THD
Harmonic-reduced bias circuit	27.07 mV	0.42 μs	37.19%	−37.68 dB	−37.83 dB	−43.80 dB	−34.82 dB
Voltage divider bias circuit	18.55 mV	0.41 μs	22.71%	−24.45 dB	−30.60 dB	−29.09 dB	−22.50 dB

**Table 5:** Comparison data between the previous publications and our scheme.

	This Work	[18]	[20]	[21]	[25]	[26]
Topology	Class-C	Class-D	Class-D	Class-DE	Class-E	Class-E
Gain	18.06 dB	--------	--------	--------	--------	--------
Output Voltage	40.0 V	--------	125 V_rms_	--------	--------	112 V_rms_
Output Power	--------	2 kW	--------	800 mW	133.3 mW	--------
Power Consumption	21.25 W	--------	--------	--------	--------	--------
Operating Frequency	25 MHz	10 kHz~100 kHz	--------	1010 kHz	41.27 kHz	28.11 kHz
HD2	−37.68 dB	--------	--------	--------	--------	--------
HD3	−37.83 dB	--------	--------	−16.40 dB	--------	--------
HD4	−43.80 dB	--------	--------	--------	--------	--------
THD	−34.82 dB	--------	--------	--------	--------	--------
Applications	Piezoelectric Transducer	Piezoelectric Transducer	Dielectric Elastomer Transducer	Piezoelectric Load	Langevin Transducer	Piezoelectric Ceramic Transducer

## Data Availability

The data presented in this study are included within the article.

## References

[1] Daniels J.M., Hoppmann R.A. (2016). Practical Point-of-Care Medical Ultrasound.

[2] Shin S.-H., Yoo W.-S., Choi H. (2019). Development of Public Key Cryptographic Algorithm Using Matrix Pattern for Tele-Ultrasound Applications. Mathematics.

[3] Moore C.L., Copel J.A. (2011). Point-of-care Ultrasonography. N. Engl. J. Med..

[4] Shin S.-H., Sok Yoo W., Choi H. (2019). Development of modified RSA algorithm using fixed mersenne prime numbers for medical ultrasound imaging instrumentation. Comput. Assist. Surg..

[5] Wagner M.S., Garcia K., Martin D.S. (2014). Point-of-care Ultrasound in Aerospace Medicine: Known and Potential Applications. Aviat. Space Environ Med..

[6] Karlen W. (2014). Mobile Point-of-Care Monitors and Diagnostic Device Design.

[7] Jeong J.J., Choi H. (2017). An impedance measurement system for piezoelectric array element transducers. Measurement.

[8] Wagner P.R., Hedrick W.R. (2014). Point-of-Care Ultrasound Fundamentals: Principles, Devices, and Patient Safety.

[9] Choi H., Li X., Lau S.-T., Hu C., Zhou Q., Shung K.K. (2011). Development of Integrated Preamplifier for High-Frequency Ultrasonic Transducers and Low-Power Handheld Receiver. IEEE Trans. Ultrason. Ferroelectr. Freq. Control.

[10] Brunner E. (2002). How ultrasound system considerations influence front-end component choice. Analog. Dialogue.

[11] Kripfgans O.D., Chan H.-L. (2021). Ultrasonic Imaging: Physics and Mechanism.

[12] Choe S.-W., Choi H. (2018). Suppression Technique of HeLa Cell Proliferation Using Ultrasonic Power Amplifiers Integrated with a Series-Diode Linearizer. Sensors.

[13] Ullah M.N., Park C., Pratiwi E., Kim C., Choi H., Yeom J.-Y. (2019). A new positron-gamma discriminating phoswich detector based on wavelength discrimination (WLD). Nucl. Instrum. Methods Phys. Res. Sect. A.

[14] Zennaro F., Neri E., Nappi F., Grosso D., Triunfo R., Cabras F., Frexia F., Norbedo S., Guastalla P., Gregori M. (2016). Real-Time Tele-Mentored Low Cost “Point-of-Care US” in the Hands of Paediatricians in the Emergency Department: Diagnostic Accuracy Compared to Expert Radiologists. PLoS ONE.

[15] Baston C.M., Moore C., Dean A.J., Panebianco N. (2019). Pocket Guide to POCUS: Point-of-Care Tips for Point-of-Care Ultrasound.

[16] Choi H., Jung H., Shung K.K. (2015). Power Amplifier Linearizer for High Frequency Medical Ultrasound Applications. J. Med. Biol. Eng..

[17] Kim J., You K., Choi H. (2020). Post-Voltage-Boost Circuit-Supported Single-Ended Class-B Amplifier for Piezoelectric Transducer Applications. Sensors.

[18] Agbossou K., Dion J.-L., Carignan S., Abdelkrim M., Cheriti A. (2000). Class D Amplifier for a Power Piezoelectric Load. IEEE Trans. Ultrason. Ferroelectr. Freq. Control.

[19] Kim K., Choi H. (2021). Novel Bandwidth Expander Supported Power Amplifier for Wideband Ultrasound Transducer Devices. Sensors.

[20] Nielsen D., Knott A., Andersen M.A.E. A high-voltage class D audio amplifier for dielectric elastomer transducers. Proceedings of the 2014 IEEE Applied Power Electronics Conference and Exposition-APEC 2014.

[21] Christoffersen C., Wong W., Pichardo S., Togtema G., Curiel L. (2016). Class-DE ultrasound transducer driver for HIFU therapy. IEEE Trans. Biomed. Circuits Syst..

[22] You K., Choi H. (2020). Wide Bandwidth Class-S Power Amplifiers for Ultrasonic Devices. Sensors.

[23] Hendee W.R., Ritenour E.R. (2003). Medical Imaging Physics.

[24] You K., Kim S.-H., Choi H. (2020). A Class-J Power Amplifier Implementation for Ultrasound Device Applications. Sensors.

[25] Yuan T., Dong X., Shekhani H., Li C., Maida Y., Tou T., Uchino K. (2017). Driving an inductive piezoelectric transducer with class E inverter. Sens. Actuators A.

[26] Niyomthai S., Sangswang A., Naetiladdanon S., Mujjalinvimut E. (2017). Operation region of class E resonant inverter for ultrasonic transducer. Proceedings of the 2017 14th International Conference on Electrical Engineering/Electronics, Computer, Telecommunications and Information Technology (ECTI-CON).

[27] Suri J.S., Kathuria C., Chang R.-F., Molinar F., Fenster A. (2008). Advances in Diagnostic and Therapeutic Ultrasound Imaging.

[28] Adhikari S., Blaivas M. (2019). The Ultimate Guide to Point-of-Care Ultrasound-Guided Procedures.

[29] Kim J., Kim K., Choe S.-H., Choi H. (2020). Development of an Accurate Resonant Frequency Controlled Wire Ultrasound Surgical Instrument. Sensors.

[30] Jung U., Choi J.H., Choo H.T., Kim G.U., Ryu J., Choi H. (2022). Fully Customized Photoacoustic System Using Doubly Q-Switched Nd: YAG Laser and Multiple Axes Stages for Laboratory Applications. Sensors.

[31] Razavi B. (2011). RF Microelectronics.

[32] Cripps S.C. (2006). RF Power Amplifiers for Wireless Communications.

[33] Zhang X., Larson L.E., Asbeck P. (2003). Design of Linear RF Outphasing Power Amplifiers.

[34] Grebennikov A. (2005). RF and Microwave Power Amplifier Design.

[35] Reynaert P., Steyaert M. (2006). RF Power Amplifiers for Mobile Communications.

[36] Kim J., You K., Choe S.-H., Choi H. (2020). Wireless Ultrasound Surgical System with Enhanced Power and Amplitude Performances. Sensors.

[37] Eroglu A. (2018). Introduction to RF Power Amplifier Design and Simulation.

[38] Eroglu A. (2017). Linear and Switch-Mode RF Power Amplifiers: Design and Implementation Methods.

[39] You K., Choi H. (2020). Inter-Stage Output Voltage Amplitude Improvement Circuit Integrated with Class-B Transmit Voltage Amplifier for Mobile Ultrasound Machines. Sensors.

[40] Albulet M. (2001). RF Power Amplifiers.

[41] Kumar N., Grebennikov A. (2015). Distributed Power Amplifiers for RF and Microwave Communications.

[42] Kazimierczuk M.K. (2014). RF Power Amplifier.

[43] Ullah M.N., Park Y., Kim G.B., Kim C., Park C., Choi H., Yeom J.-Y. (2021). Simultaneous Acquisition of Ultrasound and Gamma Signals with a Single-Channel Readout. Sensors.

[44] Choi H. (2019). Prelinearized Class-B Power Amplifier for Piezoelectric Transducers and Portable Ultrasound Systems. Sensors.

[45] Kim K., Choi H. (2021). High-efficiency high-voltage class F amplifier for high-frequency wireless ultrasound systems. PLoS ONE.

[46] Choi H. (2022). Class-C Pulsed Power Amplifier with Voltage Divider Integrated with High-Voltage Transistor and Switching Diodes for Handheld Ultrasound Instruments. Energies.

[47] Jung U., Choi H. (2022). Active echo signals and image optimization techniques via software filter correction of ultrasound system. Appl. Acoust..

[48] Choi H. (2019). Development of a Class-C Power Amplifier with Diode Expander Architecture for Point-of-Care Ultrasound Systems. Micromachines.

[49] Chen W.-K. (2002). The Circuits and Filters Handbook.

[50] Choi H. (2019). Stacked Transistor Bias Circuit of Class-B Amplifier for Portable Ultrasound Systems. Sensors.

[51] Szabo T.L. (2013). Diagnostic Ultrasound Imaging: Inside Out.

[52] Choi H., Yoon C., Yeom J.-Y. (2017). A Wideband High-Voltage Power Amplifier Post-Linearizer for Medical Ultrasound Transducers. Appl. Sci..

[53] Khan M., Khan T.M. (2022). Tunable Q matching networks for capacitive ultrasound transmitters. Analog. Integr. Circuits Signal Process..

[54] Choi H. (2022). Pre-Matching Circuit for High-Frequency Ultrasound Transducers. Sensors.

[55] Shung K.K., Smith M., Tsui B.M. (2012). Principles of Medical Imaging.

[56] Choi H. (2023). A Doherty Power Amplifier for Ultrasound Instrumentation. Sensors.

[57] Vuolevi J., Rahkonen T. (2003). Distortion in RF Power Amplifiers.

[58] Choi H., Choe S.-W. (2019). Acoustic Stimulation by Shunt-Diode Pre-Linearizer Using Very High Frequency Piezoelectric Transducer for Cancer Therapeutics. Sensors.

[59] Cripps S.C. (2002). Advanced Techniques in RF Power Amplifier Design.

[60] Larson L.E. (1996). RF and Microwave Circuit Design for Wireless Communications.

[61] Chang K. (1994). Microwave Solid-State Circuits and Applications.

[62] Irwin J.D., Wu C.-H. (1999). Basic Engineering Circuit Analysis.

[63] Choi H. (2019). Development of negative-group-delay circuit for high-frequency ultrasonic transducer applications. Sens. Actuators A.

[64] Arnau A. (2004). Piezoelectric Transducers and Applications.

[65] Kim K., Choi H. (2021). A New Approach to Power Efficiency Improvement of Ultrasonic Transmitters via a Dynamic Bias Technique. Sensors.

[66] Choi H., Jeong J.J., Kim J. (2017). Development of an Estimation Instrument of Acoustic Lens Properties for Medical Ultrasound Transducers. J. Healthc. Eng..

[67] Pasovic M., Danilouchkine M., Matte G., van der Steen A.F.W., Basset O., de Jong N., Cachard C. (2010). Broadband Reduction of the Second Harmonic Distortion During Nonlinear Ultrasound Wave Propagation. Ultrasound Med. Biol..

[68] Choi H., Woo P.C., Yeom J.-Y., Yoon C. (2017). Power MOSFET Linearizer of a High-Voltage Power Amplifier for High-Frequency Pulse-Echo Instrumentation. Sensors.

[69] Ludwig R. (2000). RF Circuit Design: Theory & Applications.

[70] Allen P.E., Holberg D.R. (2002). CMOS Analog Circuit Design.

[71] Choi H., Park C., Kim J., Jung H. (2017). Bias-Voltage Stabilizer for HVHF Amplifiers in VHF Pulse-Echo Measurement Systems. Sensors.

[72] Pederson D.O., Mayaram K. (2007). Analog Integrated Circuits for Communication: Principles, Simulation and Design.

[73] Zawawi R.B.A., Choi H., Kim J. (2020). High-PSRR Wide-Range Supply-Independent CMOS Voltage Reference for Retinal Prosthetic Systems. Electronics.

[74] Grebene A.B. (2002). Bipolar and MOS Analog Integrated Circuit Design.

[75] Zawawi R.B.A., Abbasi W.H., Kim S.-H., Choi H., Kim J. (2020). Wide-Supply-Voltage-Range CMOS Bandgap Reference for In Vivo Wireless Power Telemetry. Energies.

[76] Chen W.-K. (2003). Analog Circuits and Devices.

[77] Choi H., Choe S.-W. (2018). Therapeutic Effect Enhancement by Dual-bias High-voltage Circuit of Transmit Amplifier for Immersion Ultrasound Transducer Applications. Sensors.

[78] Johns D.A., Martin K. (2008). Analog Integrated Circuit Design.

[79] Choi H. (2023). An Inverse Class-E Power Amplifier for Ultrasound Transducer. Sensors.

[80] Grebennikov A., Sokal N.O., Franco M.J. (2011). Switchmode RF Power Amplifiers.

[81] Gray P.R. (2009). Analysis and Design of Analog Integrated Circuits.

[82] Razavi B. (2016). Design of Analog CMOS Integrated Circuits.

[83] Carr J. (2002). RF Components and Circuits.

[84] Lee T.H. (2006). The Design of CMOS Radio-Frequency Integrated Circuits.

[85] Hong J., Oh Y., Choi H., Kim J. (2022). Low-Area Four-Channel Controlled Dielectric Breakdown System Design for Point-of-Care Applications. Sensors.

[86] Abbasi W., Choi H., Kim J. (2022). Hexagonal Stimulation Digital Controller Design and Verification for Wireless Subretinal Implant Device. Sensors.

[87] Colantonio P., Giannini F., Limiti E. (2009). High Efficiency RF and Microwave Solid State Power Amplifiers.

[88] Kang H., Choi H., Kim J. (2021). Ambient Light Rejection Integrated Circuit for Autonomous Adaptation on a Sub-Retinal Prosthetic System. Sensors.

[89] Zawawi R.B.A., Choi H., Kim J. (2021). High PSRR Wide Supply Range Dual-Voltage Reference Circuit for Bio-Implantable Applications. Electronics.

[90] Kim J., Kim K.S., Choi H. (2021). Development of a low-cost six-axis alignment instrument for flexible 2D and 3D ultrasonic probes. Technol. Health Care.

[91] Choi H. (2022). Novel dual-resistor-diode limiter circuit structures for high-voltage reliable ultrasound receiver systems. Technol. Health Care.

[92] Shutilov V.A., Alferieff M.E. (2020). Fundamental Physics of Ultrasound.

[93] Choi H., Yang H.-C., Shung K.K. (2014). Bipolar-power-transistor-based limiter for high frequency ultrasound imaging systems. Ultrasonics.

[94] Shung K.K. (2015). Diagnostic Ultrasound: Imaging and Blood Flow Measurements.

[95] Choi H., Ryu J.-M., Choe S.-W. (2019). A novel therapeutic instrument using an ultrasound-light-emitting diode with an adjustable telephoto lens for suppression of tumor cell proliferation. Measurement.

[96] Self D. (2013). Audio Power Amplifier Design.

[97] Safari A., Akdogan E.K. (2008). Piezoelectric and Acoustic Materials for Transducer Applications.

